# An inherited *SLC25A12*-related recessive form of congenital porencephaly in Limousin cattle

**DOI:** 10.1186/s12711-026-01057-6

**Published:** 2026-06-07

**Authors:** Joana Jacinto, Tobias Floyd, Anna Letko, Heather Stevenson, Vanessa Swinson, Helen Carty, Irene M. Häfliger, Franz R. Seefried, Cécile Grohs, Mekki Boussaha, Ben Strugnell, Beverley Hopkins, Arthur Otter, Aurélien Capitan, Cord Drögemüller

**Affiliations:** 1 Clinic for Ruminants, University of Bern, 3012 Bern, Switzerland; 2 Institute of Genetics, University of Bern, 3012 Bern, Switzerland; 3 Animal and Plant Health Agency, Thirsk, YO7 1PZ UK; 4 SRUC Veterinary and Analytical Services, Pentlands Science Park, Bush Estate Loan, Penicuik, Midlothian, EH26 0PZ UK; 5 Qualitas AG, Zug, Switzerland; 6 GABI, INRAE, AgroParisTech, Université Paris-Saclay, 78350 Jouy-en- Josas, France; 7 Farm Post Mortems, Hamsterley House, Hamsterley, Bishop Auckland, County Durham, UK; 8 Wales Veterinary Science Centre, Y Buarth, Aberystwyth, Ceredigion, SY23 1ND Wales, UK

## Abstract

**Background:**

Congenital abnormalities in cattle, which include lesions in the central nervous system, usually occur sporadically. Porencephaly, a condition characterised by the presence of cystic fluid-filled cavities within brain tissue, is often virus-induced, but rare inherited forms have been identified in other species. Thirty-four calves affected by porencephaly were reported in Limousin cattle in Great Britain and 16 underwent clinicopathological investigation. We aimed to: (1) characterize the disorder phenotype, (2) investigate its possible genetic cause, and (3) determine the frequency of the identified variant across Limousin populations.

**Results:**

Affected calves presented blindness and stupor from birth and were unable to suckle without assistance. Brain examination revealed a bilateral symmetrical cavity in the cerebral cortex (porencephaly). Some of the affected calves also showed evidence of ongoing degeneration of the cerebellar cortex, typified by focal, spindle-shaped swellings found on the proximal axons of Purkinje cells (known as ’torpedoes’) within the inner granular layer and intra-myelinic phagocytes in the white matter of the cerebellar folia (cerebellar abiotrophy). After PCR-based exclusion of common teratogenic viruses, monogenic recessive inheritance was hypothesized based on pedigree analysis. Whole-genome mapping and sequencing approaches identified a unique homozygous genome region of 1.2 Mb on chromosome 2 with a private homozygous missense variant in *SLC25A12* (NM_001101194.2: c.1742G > A; NP_001094664.1:p.(Arg581Gln)) in three cases. This variant was absent in > 5,000 control genomes. The affected gene encodes a calcium-binding mitochondrial carrier protein and is a known candidate for neurogenetic disorders. Genotyping confirmed recessive inheritance in the random study cohort of British Limousins, with the *SLC25A12* variant having an allele frequency close to 0% in the studied French and Swiss Limousin populations.

**Conclusions:**

We report the first *SLC25A12*-related neurodevelopmental disorder in a domestic animal species and assume that the identified pathogenic variant impairs the normal function of the SLC25A12 protein. Thereby, this study provides a new spontaneous large animal model for similar human conditions. The identified allele should be considered in cattle breeding programs to prevent risk matings. Since several neurogenetic disorders share a morphology with virus-induced congenital malformations, diagnostic virus testing should always be considered for aborted, stillborn or live-born calves with porencephaly, alongside a possible genetic aetiology.

**Supplementary Information:**

The online version contains supplementary material available at 10.1186/s12711-026-01057-6.

## Background

Several syndromic and non-syndromic forms of congenital central nervous system (CNS) abnormalities affecting the brain, which belong to the heterogenous group of neurodevelopmental disorders, are known to occur in cattle. These are characterised by cerebellar abiotrophy (OMIA 000175–9913), cerebellar cortical atrophy (OMIA 000177–9913), cortical cerebellar disease (OMIA 000178–9913), cerebellar hypoplasia (OMIA 000179–9913), hydranencephaly and cerebellar hypoplasia (OMIA 001689–9913), hydranencephaly (OMIA 000486–9913), hydrocephalus (OMIA 000487–9913), lissencephaly (OMIA 002771–9913), and porencephaly (OMIA 003026–9913). These potentially genetic disorders, which exhibit partially overlapping features, share morphological characteristics with developmental conditions caused by teratogenic viruses and may be associated with skeletal abnormalities that contribute to dystocia [1]. Therefore, aetiology diagnosis of these sporadic conditions, which typically involve mostly unknown pathogenetic mechanisms, is challenging [2].

Congenital porencephaly is a rare condition affecting the CNS that has been described in humans and domestic animals including cattle [3–7]. It is characterized by cystic fluid filled cavities in the forebrain neocortex which result from destruction and maldevelopment of the neocortex during embryogenesis [8, 9]. Porus cavities within white matter may be single or multiple and randomly or symmetrically distributed [3, 9]. They may be accompanied in the neocortex by thinning and abnormal gyration of the grey matter and thinning of white matter tracts, including the corpus callosum. In some cases, through loss of tissue, porous cavities may communicate with the lateral ventricles or the subarachnoid space [3]. The brainstem and hindbrain may or may not be concurrently affected, depending on the cause. Porencephaly can be considered as part of a spectrum of destruction and loss of cerebral tissue that includes hydranencephaly (where there is complete destruction of the neocortex) in its most severe form [8]. Porencephaly may result from destruction and loss of neuroblasts during brain development, vascular injury to the developing brain, or a combination of the two [4, 6, 10].

In domestic ruminants (including cattle, sheep and goats), porencephaly is most seen following intrauterine infection with certain teratogenic viruses [2]. Among these, bovine viral diarrhoea virus (BVDV), border disease virus (BDV), bluetongue virus (BTV) and Simbu serogroup *Orthobunyaviruses* such as Schmallenberg virus (SBV) are currently prevalent in northern Europe [11–17]. However, porencephaly may also be occasionally seen in lambs suffering copper deficiency *in utero* (swayback) [9]. In humans, in addition to intrauterine viral infections [18], there are several reported conditions involving porencephaly where a genetic basis is supposed or confirmed (OMIM601322, OMIM175780, OMIM614483, OMIM618360). In these rare monogenic disorders, where the causal variants are well characterised, defects in collagen synthesis often lead to increased fragility of small blood vessels in the brain and other tissues [19]. There are also reports of syndromic forms of congenital porencephaly in lambs and water buffalo where genetic causes were suspected but not further characterised [20, 21].

Between 2020 and 2025, 34 Limousin calves across England, Scotland and Wales were documented with a consistent clinicopathological phenotype, involving congenital porencephaly. This consistent clinicopathological phenotype, along with analysis of breeding records suggested a genetic aetiology.

Therefore, the aim of our study was: (1) to characterize the clinicopathological findings of this congenital disorder in Limousin cattle, (2) to investigate the possible genetic cause using SNP array genotyping and whole-genome sequencing (WGS) data analyses, and (3) to determine the frequency of the identified variant across different European Limousin populations.

## Methods

### Animals, testing for prevalent teratogenic viruses, and clinicopathological investigation

This study did not require official or institutional ethical approval as it was not experimental, but rather part of routine clinical veterinary diagnostics. All animals in this study were examined with the consent of their owners and handled according to recognized ethical standards.

Between 2020 and 2025, investigators from the Animal and Plant Health Agency (APHA), the Scottish Rural University Colleges (SRUC), and associated surveillance pathology partners received reports of 34 affected calves from farms across England, Scotland, and Wales, of which 16 were clinicopathologically examined. A full post-mortem examination was performed on the 16 calves and samples of selected tissues taken for further histological assessment and ancillary testing, as deemed appropriate.

Detailed pathological examination of the fixed brain was performed on all 16 calves. Other tissues examined included eye, spinal cord, skeletal muscle, heart, lung, liver, kidney, spleen and thyroid. Tissues were fixed in 10% neutral buffered formalin and processed by routine histological methods. Four-micron thick sections of tissues were stained with haematoxylin and eosin for microscopic examination.

To investigate potential viral aetiologies, PCR testing for BTV, BVDV and BDV on spleen and SBV on cerebrum of the 16 clinicopathologically examined calves was performed in the APHA or SRUC.

#### Pedigree records and DNA samples

Pedigree records for 24 of the 34 reported Limousin cases were obtained from the breeders and the British Limousin Cattle Society. In addition, pedigrees of their earliest French Limousin ancestors were retrieved from the French national bovine database identification [22]. From these records, a pedigree was constructed using Pedixplorer v1.6.0 [23].

Out of the 16 pathologically examined calves, biological material (ear tissue) was available from three cases, including two paternal half-siblings (cases 1 and 2) and a third, more distantly related case (case 3). In addition, EDTA-blood samples were available from the single sire and two dams of cases 1 and 2, as well as from 58 unrelated animals representing the British Limousin population. Genomic DNA was extracted from ear tissue and EDTA-blood samples using Promega Maxwell RSC DNA system (Promega, Dübendorf, Switzerland).

#### Whole-genome sequencing (WGS)

WGS data was generated using the Illumina NovaSeq6000 platform (Illumina Inc., San Diego, CA, USA) from four genomic DNA samples including three porencephaly-affected calves (cases 1 to 3) and one available sire. The sequenced reads were mapped to the ARS-UCD1.2 reference genome [24], resulting in an average read depth of 21×, and single-nucleotide variants (SNV) and small indels were called. The applied software and steps to process fastq files into binary alignment map and genomic variant call format (VCF) files were in accordance with the 1000 Bull Genomes Project processing guidelines of run 7 [25], except for the trimming, which was performed using fastp [26]. Downstream processing of the genomic data was performed as reported previously [27]. The effects of all called variants were functionally evaluated with snpEff v5.0c [28], using the NCBI Annotation Release 106 (https://www.ncbi.nlm.nih.gov/genome/annotation_euk/Bos_taurus/106/). This resulted in the final VCF file, comprising jointly genotyped individual variants of 1038 animals including the three cases, the sire, and 1034 diverse controls from the ongoing Swiss Comparative Bovine Resequencing project including 38 Limousin including the three cases (European Nucleotide Archive: project accession numbers PRJEB18113 and PRJEB83441). These Limousin controls originated from Switzerland, UK, Denmark, France, Italy and Germany.

#### Homozygosity analyses

The three affected calves and the obligate carrier bull were genotyped using the Illumina EuroGMD SNP array [29]. Their genotypes were analyzed, following the French genomic evaluation workflow, together with those of 19,857 healthy Limousin individuals genotyped in France over the past decade as part of routine genomic evaluations. This workflow includes parentage verification, haplotype phasing, and imputation to the BovineSNP50 panel using FImpute3 [30], as described by Mesbah-Uddin et al. [31]. Marker positions were based on the ARS-UCD1.2 bovine genome assembly. A homozygosity mapping approach was then performed using sliding haplotypes of 20 markers (~ 1 Mb), as described by Boulling et al. [32]. Briefly, Fisher’s exact tests were conducted comparing the number of animals homozygous for a given haplotype to those carrying any other genotype between case and control groups. A total of 210,184 haplotypes were analysed. To account for multiple testing, a Bonferroni-corrected cut-off at *p* = 0.01 was applied, adjusted as −log₁₀P = −log₁₀((0.01/210,184)×20) = 6,02, reflecting partial non-independence of overlapping 20-marker windows. Adjacent overlapping haplotypes for which all cases and none of the controls were homozygous were used to define the mapping interval. Finally, a haplotype test involving 23 contiguous markers was applied to predict the status at the porencephaly-associated locus within the control group.

WGS-based runs of homozygosity (ROH) analysis was performed on the 49,965,295 variants called in all 38 purebred Limousin genomes extracted from the Swiss Comparative Bovine Resequencing project. PLINK v1.9 [33] was used for quality control pruning: only high-quality biallelic SNVs mapped to the 29 bovine autosomes called in all individuals with minor allele frequency > 0.05 and Hardy-Weinberg equilibrium exact test *p*-value >1 × 10^− 6^ were retained for the downstream analyses, which constituted a set of 11,785,038 SNVs. Genome-wide search for ROH in regions shared by the three calves was performed using the R package detectRUNS v.0.9.6 [34]. Based on published guidelines [35], the following parameters were set for the ROH detection: sliding window size of minimum 20 SNVs, maximum of 3 heterozygous genotypes in a window, minimum ROH length of 200 kb, minimum number of 66 SNVs in a ROH, and minimum density of one SNV per 50 kb [36]. Additionally, homozygosity-based genomic inbreeding coefficient (F_ROH) was calculated based on the total length of the autosomal genome covered by SNV positions (herein 2,488,159,091 bp) and the F_ROH of the three studied cases were compared to the mean F_ROH obtained from the analysis of the 34 control Limousin genomes (Additional File 1 Table S1).

### Candidate gene and candidate variant classification

A candidate gene was defined as a gene selected based on its known function and/or reported association with neurodevelopmental phenotypes in mammals, as determined from a literature survey. A candidate variant was defined as a rare variant affecting a candidate gene and predicted to have a deleterious impact on the encoded protein by at least three in silico prediction tools. In addition, the identified protein-changing variant was further classified according to established guidelines for the interpretation of sequence variants in human and animal genetics [37, 38].

#### Search for candidate variants and in silico assessment of their molecular consequences

Within the identified ROH interval on chromosome 2 from position 24,528,003 to 25,722,296 bp, the annotated genotypes of the three affected calves and the sire were compared to 1,927 control genomes sequenced as part of successive resequencing projects in Switzerland (*n* = 1,031) [39] and France (*n* = 895, Additional File 2 Table S2) [40–42]. This dataset comprised genomes from 124 Limousin individuals, all predicted to be homozygous wild type based on haplotype, as well as genomes from more than 100 other breeds.

We performed variant filtering under the hypothesis of a recessive, breed-specific genetic defect. Accordingly, we assumed that all three affected individuals would be homozygous for the variant allele, the available sire would be obligate heterozygous, and the controls would be either heterozygous or homozygous for the reference allele.

After filtering, PredictSNP1 [43], PolyPhen-1, Polyphen-2 [44], SIFT [45], PhD-SNPg [46], and MutPred2 [47] were used to predict the biological consequences of the only remaining candidate variant in *SLC25A12*. Additionally, IGV software version 2.0 [48] was used for visual inspection of the candidate variant resulting from variant filtering.

#### Occurrence of the ***SCL25A12*** variant in a global control cohort

The comprehensive variant catalogue from run 9 of the 1000 Bull Genomes Project was available to investigate the allelic distribution of the identified candidate variant in *SCL25A12* within a global control cohort [25]. The full dataset comprised 5116 bovine genomes, including 576 from the Swiss Comparative Bovine Resequencing Project, from a wide variety of more than 130 breeds.

### Targeted genotyping of the SCL25A12 variant

PCR and Sanger sequencing were used to validate and genotype the *SLC25A12* missense variant in three cases and an obligate carrier sire and two dams. Additionally, a random cohort of 58 Limousin individuals from the UK, opportunistically sampled from farms with reported cases, were genotyped using the same method. PCR products from genomic DNA were amplified using AmpliTaq Gold 360 Master Mix (Thermo Fisher Scientific, Waltham, MA, USA) and the PCR amplicons were directly sequenced on an ABI3730 capillary sequencer (Thermo Fisher Scientific, Darmstadt, Germany). The *SLC25A12* missense variant was genotyped using the following primers: 5′-CCCTCAGCATTTTGGAAAGGA-3′ (forward primer) and 5′-TGTCCTGTCGGTTGCTATCC-3′ (reverse primer).

The identified protein-changing variant in *SCL25A12* was added to the subsequently updated versions of the Swiss Axiom custom genotyping arrays (Thermo Fisher Scientific, Waltham, MA, USA) routinely used for genomic selection in Switzerland. Thus, after conducting population-wide genotyping of Swiss Limousin cattle for genomic selection purposes, several hundred genotypes for the variant were available. Subsequently, the prevalence of the identified *SCL25A12* allele was evaluated.

## Results

### Clinicopathological indication of the presence of a novel form of porencephaly with cerebellar atrophy

From the 16 clinicopathologically examed calves, 15 calves were born alive, and one was aborted at the third trimester of gestation. All liveborn calves were euthanised shortly after birth due to poor viability, except one that survived to 10 weeks of age but ultimately died.

The 15 liveborn calves exhibited clinical signs from birth, including generalized weakness, blindness, reduced mentation, and compulsive wandering. Although the suckling reflex was present, all calves required assistance with feeding. The single calf that survived to 10 weeks demonstrated progressive neurological deterioration, with additional signs including cerebellar ataxia.

Tissue samples from all affected calves tested negative for BVDV, SBV, and BTV using PCR. These findings likely rule out intrauterine viral infections with these currently prevalent agents as the cause of the observed phenotype.

**Fig. 1:**
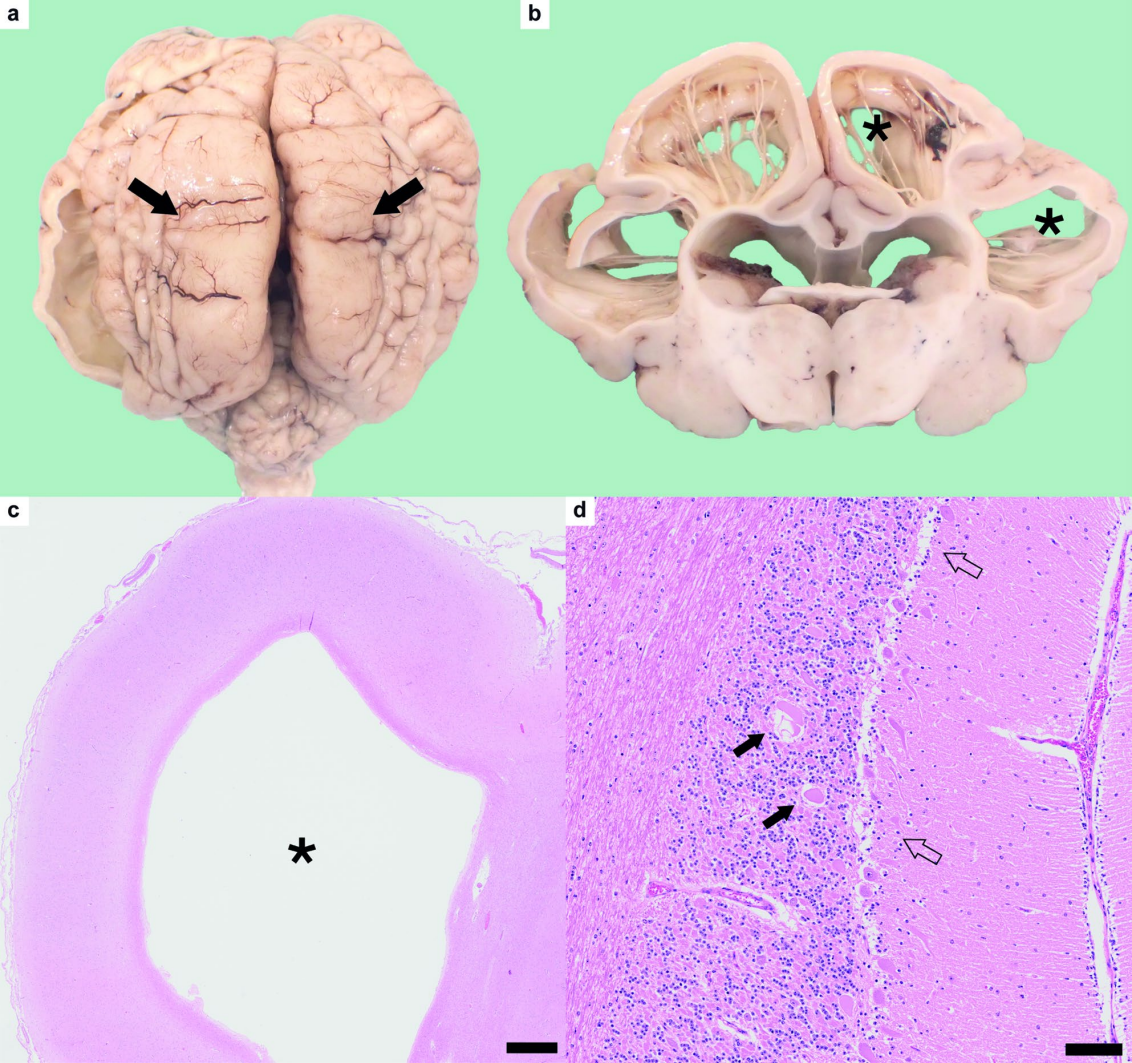
Brain pathological findings in a Limousin calf affected by porencephaly. **a** Dorsal view of formalin-fixed whole brain showing bilaterally similar lissencephaly of the parietal cortex (black arrows). **b** Coronal section of formalin-fixed cerebrum and thalamus showing bilaterally similar porencephaly of the cerebral white matter (asterisks). **c** Photomicrograph of a typical smooth-walled porus cavity in the cerebral white matter (asterisk) lined by astrocyte processes. Haematoxylin and eosin. Bar = 1 mm. **d** Photomicrograph of the cerebellar cortex of an affected calf which lived to 10 weeks of age showing degeneration of the cerebellar cortex in the form of segmental swelling and vacuolation of axons in the internal granular layer (torpedos formation; black arrows) and multifocal loss of Purkinje neurons (open arrows). Haematoxylin and eosin. Bar = 100 microns

The main gross pathological finding in the 16 calves examined was extensive, bilaterally similar porencephaly and lissencephaly of the cerebral cortex (Fig. 1a, b). Smooth-walled porus cavities, traversed by diaphanous vessels, were present in the white matter throughout the frontal, parietal and occipital cortices and were accompanied by dilation of the lateral ventricles (hydrocephalus ex vacuo), thinning of the corpus callosum and thinning and abnormal gyration of the cerebral grey matter. The brainstem and cerebellum were grossly unaffected. One calf examined additionally had a purulent exudate over the meninges and within the ventricular system, indicative of a bacterial meningitis.

The whole fixed brains of the calves examined varied in weight from 169 g to 229 g (median 200 g) and the cerebella from 20 g to 25.5 g (median 24 g). The brain weight of the aborted calf and one live-born calf which was shot dead were excluded from this assessment.

Microscopic examination of the porus lesions revealed smooth-walled cavities lined by glial cells in the cortical white matter (Fig. 1c). The cerebral cortical grey matter was reduced in thickness and neuronal populations were subjectively depleted and the laminae were poorly organised. Microscopic examination of the cerebellar cortex revealed Purkinje neuron degeneration, necrosis and loss, accompanied by swollen proximal axonal segments in the internal granular layer (torpedoes; Fig. 1d). Axonal swelling and Wallerian-type degeneration was also evident in the cerebellar white matter. The severity of degenerative changes in the cerebellar cortex varied between the calves examined. Changes were very subtle to non-existent in a few cases, but comparatively quite severe in the calf which had survived to 10 weeks of age. Samples of skeletal muscle examined from two affected calves showed mild variation of myofibre diameter, with some smaller fibres showing hyalinisation of the sarcoplasm. All other tissues were unremarkable on microscopic examination.

Based on the clinicopathological findings the affected Limousin calves were diagnosed with a form of porencephaly.

### Pedigree analysis implies monogenic recessive inheritance

Multiple calves were affected within individual herds, and cases occurred across at least three calving seasons, which suggests that the cause was a persistent and possibly inherited. Within herd incidence of cases varied, with some herds reporting only one affected calf and others reporting multiple affected calves over successive breeding seasons. Affected farms also reported unconfirmed fertility problems, including early embryonic losses, abortions and stillbirths.

**Fig. 2:**
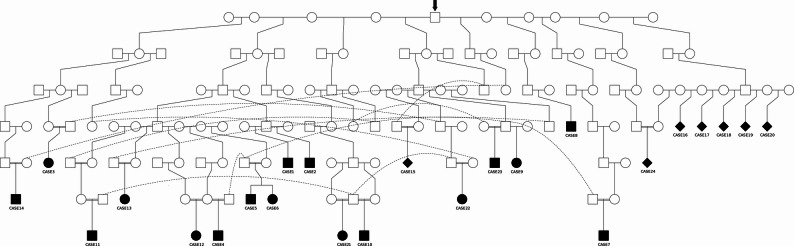
Pedigree analysis of the 24 porencephaly-affected calves with available records. Pedigree analysis revealed that 24 cases of both sexes (black filled shapes) were related to a common ancestor within six generations (black arrow) indicating autosomal recessive mode of inheritance. Note the cross-generational matings and inbreeding loops leading to several individuals plotted repeatedly for the sake of readability (identical animals are shown as symbols connected with dashed lines)

Pedigree analysis of the 24 affected calves, for which pedigree records were available, revealed a common male ancestor within six generations, suggesting a shared genetic background that could indicate a monogenic autosomal recessive mode of inheritance (Fig. 2). The shared ancestor (named “Sy”) was born in France in 2001 and was later exported to the UK, where it became a popular artificial insemination bull.

#### Mapping to a critical homozygous genome region of ~ 1.2 Mb on chromosome 2

**Fig. 3:**
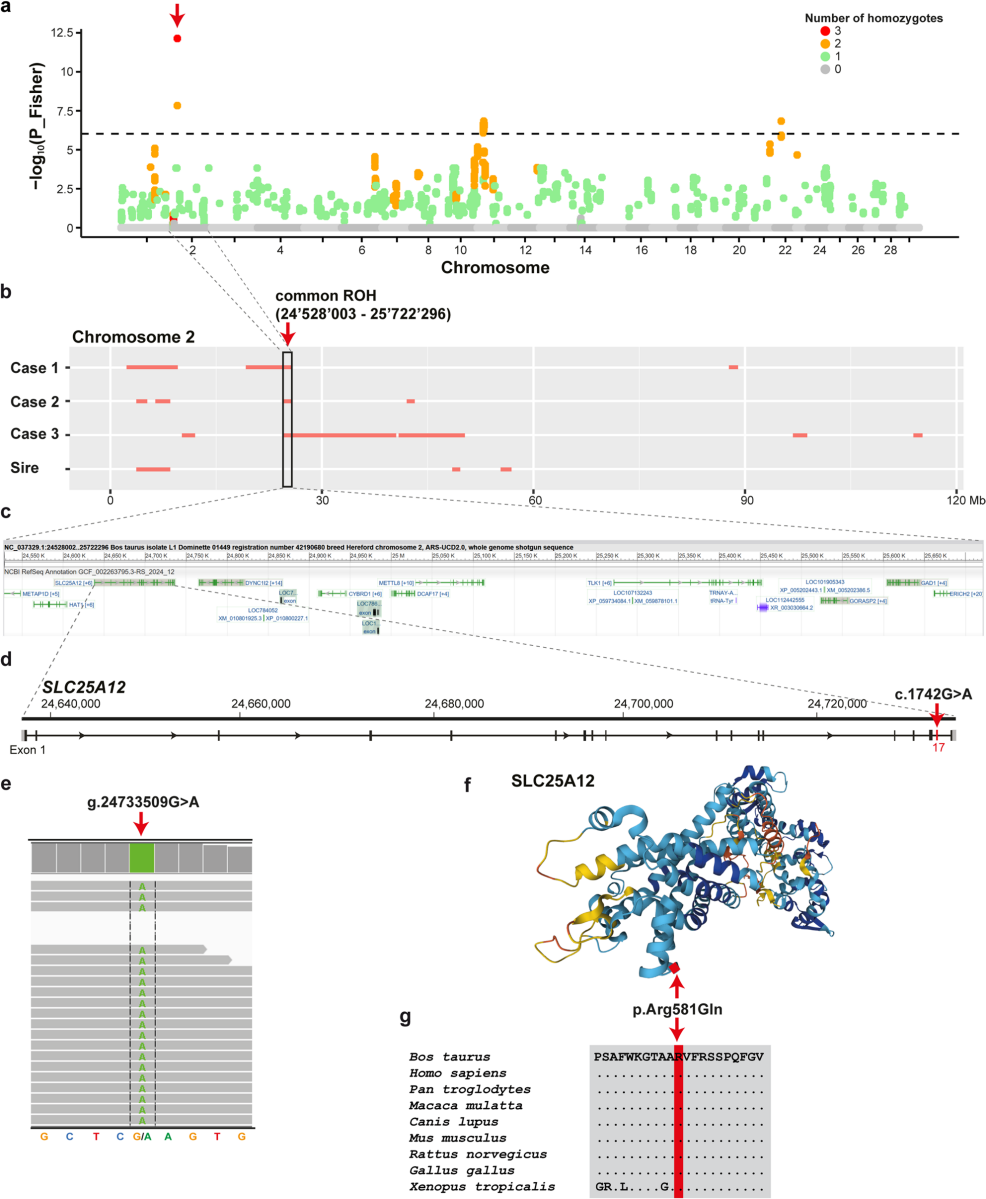
Mapping of an associated homozygous genome region on cattle chromosome 2 and identification of a ***SLC25A12*** missense variant. **a** Results of Fisher’s exact tests comparing the proportion of homozygous carriers for 20-marker sliding haplotypes (with one-marker step) between 3 affected calves and 19,857 controls. To account for multiple testing, a Bonferroni-corrected cut-off at *p* = 0.01 was applied, corresponding to −log₁₀*P* = 6.02 (dashed horizontal line). Points are coloured according to the number of homozygotes for each haplotype in the affected calves’ group (Ahmz): red for Ahmz = 3, orange for Ahmz = 2, light green for Ahmz = 1, and grey or light grey for the remaining haplotypes with Ahmz = 0 depending on chromosome parity. A peak on chromosome 2 spans four overlapping windows, forming a merged 23-marker haplotype, homozygous in the three cases and none of the controls (raw *p* = 7.66 × 10^−13^ Bonferroni-corrected *p* = 8.05 × 10^−9^). **b** Plot of runs of homozygosity (ROH) on chromosome 2 shared by the three affected calves. Homozygous regions are shown as pale red segments along the graph. The genomic consensus homozygous region is marked with a red arrow and framed in a black rectangle. It spans from 24’528’003 to 25’722’296 (approx. 1.2 Mb) and contains 19 annotated genes. **c** Screenshot from the NCBI Genome Data Viewer showing the ROH on chromosome 2, illustrating the 19 annotated genes located within this interval. **d ***SLC25A12* gene structure showing the location of the variant NM_001101194.2:c.1742G > A on chromosome 2, exon 17 (red arrow). **e** Integrative genomic viewer screenshot showing the CM008169.2:g.24733509G > A variant in the cases revealed by WGS. **f** Schematic representation of the bovine SLC25A12 protein. The position of the NP_001094664.1:p.(Arg581Gln) variant is indicated by the red arrow. **g** Multiple sequence alignment of the SLC25A12 protein encompassing the region of the affected residue reveals complete evolutionary conservation across species

Assuming that this bovine form of porencephaly represents a Mendelian disorder, we first applied a homozygosity mapping approach. We analysed the imputed and phased Illumina BovineSNP50 genotypes of three affected calves and 19,857 controls, using sliding windows of 20 markers and identified a single critical genome segment (Fig. 3a). Within the peak region on chromosome 2, we identified a 23-marker haplotype, which was homozygous in all affected calves and absent in all controls. The most proximal markers outside of this segment defined the boundaries of a ~ 1.3 Mb critical interval on chromosome 2 from position 24,526,774 to 25,816,888 bp.

In a second step, ROH analyses were performed using the WGS data of the Swiss Limousin dataset (*n* = 38). Genomic inbreeding coefficient (F_ROH) was estimated based on the detected ROH (Additional file 1 Table S1). F_ROH of the three affected calves was 0.17 (case 1), 0.14 (case 2), and 0.17 (case 3), the sire of case 1 and 2 showed a F_ROH of 0.19. Overall, these were slightly higher than the mean of the studied Swiss control cohort (mean 0.16, SD ± 0.03, range 0.05–0.22; Additional file 1 Table S1).

The three affected Limousin calves shared 130 regions of homozygosity. Only one of these exceeded 1 Mb, coinciding with the previously detected homozygosity mapping interval on chromosome 2 (Fig. 3b, Additional File 3 Table S3). This ROH analysis enabled fine mapping of the critical interval to a region spanning ~ 1.2 Mb, from 24,528,003 to 25,722,296 bp, and containing 19 annotated genes (Fig. 3b, Additional File 4 Table S4).

#### Identification of a pathogenic missense variant in ***SLC25A12***

Subsequently, the established SNV data were filtered for candidate causal variants. Within the consolidated mapping interval on chromosome 2, a total of nine sequence variants were identified for which the three affected calves were homozygous for the variant allele and the obligate carrier sire was heterozygous (Additional File 5 Table S5). No structural variants or small indels were identified within the candidate region. Then filtering out variants found in 124 control Limousin predicted to be homozygous wildtype based on a haplotype test and 1,927 additional controls from more than 100 breeds, reduced the list of candidate variant to a single missense variant located in exon 17 of *SLC25A12* (CM008169.2:g.24733509G > A; NM_001101194.2: c.1742G > A), which was confirmed by visual inspection and subsequent Sanger sequencing (Fig. 3c–e). This *SLC25A12* missense variant was predicted to alter the encoded amino acid residue at position 581 of SLC25A12 (NP_001094664.1: p.(Arg581Gln); Fig. 3f–g), located within the mitochondrial carrier domain. In addition, the variant was predicted to be deleterious by most of the used in silico tools and the affected amino acid was highly conserved across species revealing complete evolutionary conservation (Fig. 3g). The *SLC25A12* variant was classified as pathogenic (Additional file 6, Table S6). This variant was added to OMIA database under the accession number OMIA:003026–9913: Porencephaly, *SLC25A12*-related in *Bos taurus* (taurine cattle) with the OMIA variant ID:1853.

#### The harmful ***SLC25A12*** allele originates in France

The presence of the *SLC25A12* variant was investigated using genotyping data from a global population of Limousin cattle and other breeds included in the 1000 Bull Genomes Project. No heterozygous nor homozygous mutant animals were identified in this cohort, suggesting that the variant is either extremely rare or absent from the broader global population.

Additionally, the prevalence of the *SLC25A12* variant was evaluated in opportunistically sampled Limousin controls from farms with reported cases and two other European Limousin populations (Swiss and French) using direct genotyping or inference from a haplotype test for the French animals (Table 1). The French and Swiss cohorts showed markedly low allele frequencies, with a frequency of 0.0003 observed in the French cohort and no variant alleles detected in the Swiss cohort. Among the 19,857 animals genotyped for routine genomic evaluation in France, only 13 heterozygous carriers of the at-risk haplotype were identified, most of whom were related to the paternal granddam of “Sy”.

**Table 1:** Occurrence of the *SLC25A12* variant in different Limousin cattle cohorts

Cohort	Ref/ref	Ref/var	Var/var
Global cattle*	5279	0	0
British Limousin	37	21**	3***
Swiss Limousin	230	0	0
French Limousin****	19,844	13	0

*Run 9 of 1000 Bull Genomes Project including 101 purebred Limousin; ** including 3 obligate carriers *** cases 1 to 3; **** indirect haplotype-based detection

## Discussion

We present a description of the clinical and pathological features of Limousin calves affected by congenital CNS abnormalities including porencephaly with lissencephaly and cerebellar abiotrophy. The affected calves were mostly born alive and showed congenital clinical signs, including generalised weakness, blindness and compulsive wandering, with some calves showing progressive cerebellar signs, including cerebellar ataxia. The combination of established porous lesions in the cerebrum and ongoing neuronal degeneration in the cerebellum suggested an intrinsic error of cellular metabolism. Based on the consistent phenotype, negative results from testing for teratogenic viral agents, and subsequent pedigree analyses, we hypothesized that the disorder was caused by a harmful monogenic recessive allele. Subsequently, we performed homozygosity mapping and whole genome sequencing that resulted in the identification of a single homozygous protein changing variant in a candidate gene that was shared by the three sequenced cases. After analysing gene function and considering the rarity of the variant, its co-segregation in several affected individuals, in silico predictions of its impact, and the conservation of the affected amino acid, we determined that the identified missense variant in *SLC25A12* is pathogenic [38] and represents the genetic cause of the observed congenital condition. Therefore, we provide the very first example of a precise aetiologic diagnosis of a novel neurodevelopmental disorder among various reported congenital CNS disorders in cattle.

The identification of a pathogenic missense variant in *SLC25A12* in affected Limousin calves provides a plausible molecular explanation for the observed phenotype, particularly considering the gene’s well-characterized role in mitochondrial function and neuronal metabolism. *SLC25A12* encodes the electrogenic aspartate/glutamate antiporter SLC25A12, a carrier protein on the inner mitochondrial membrane which forms part of the malate-aspartate shuttle (MAS) [49–53]. Additionally, this protein is highly expressed in excitable tissues of the brain, heart and muscle [53]. The MAS transports reducing equivalents across the inner mitochondrial membrane, maintaining the cytosolic and intramitochondrial redox balance and linking cytosolic glycolysis with the tricarboxylic acid (TCA) cycle and the electron transport chain (ETC), thereby supporting efficient mitochondrial respiration [53, 54]. The MAS also produces glutamate and aspartate, amino acids with vital roles in synthesis of other amino acids, proteins, nucleic acids and neurotransmitters [53, 54]. Its importance to the central nervous system is highlighted by the fact that most intrinsic disorders of the MAS recognised in humans affect the central nervous system with onset in early life [53, 54].

Pathogenic variants in *SLC25A12* have been identified and studied at molecular level in different species including dogs (OMIA 002294–9615), humans (OMIM 603667), and mice (MGI 1926080).

In dogs, two independent missense variants have been reported to cause recessive forms of cerebellar degeneration-myositis complex in Dutch Shepherd and Nova Scotia Duck Tolling Retriever dogs [55, 56]. In both breeds, the neurodevelopmental disorder is characterized by early-onset and progressive muscle weakness, tremors, stiffness, and muscle atrophy, with elevated serum creatine kinase levels indicating myofiber damage. Investigation of the condition in Dutch Shepherd dogs showed that impaired AGC1 function led to oxidative stress and elaboration of a milieu of pro-inflammatory metabolites within muscle cells [56]. Affected homozygous Dutch Shepherds did not display neurological signs, and the nervous system was not examined [56]. The condition in Nova Scotia Duck Tolling Retrievers, however, did involve bilaterally symmetrical degeneration of nuclei in the cerebellum, leading the authors to name the condition cerebellar degeneration-myositis complex, but they provide little further detail of the pathology in these dogs [55].

In humans, developmental and epileptic encephalopathy 39 with leukodystrophy (DEE39, OMIM 612949), is a disorder characterised by early onset developmental delay, seizure and hypotonia [57]. Magnetic resonance imaging (MRI) of subjects reveals progressive atrophy of the brain and hypomyelination of the cerebral white matter [57]. Decreased levels of aspartate and N-acetyl aspartate in brain and elevated serum lactate have been demonstrated in subjects, in line with dysfunction of AGC1. As with other inborn errors of metabolism, DEE is particularly rare, with only 16 cases having been described to date [54]. In most cases where the genetic basis has been characterised, homozygosity for a missense variant in the *SLC25A12* has been identified, resulting in profound dysfunction of AGC1.

Interestingly, the clinical and pathological effects of AGC1 dysfunction seen in human patients were predicted through experiments involving knockout mice several years before the first human case was recognised [58]. Epileptic activity is thought to arise from impaired neuronal energy metabolism and impaired production and cycling of neurotransmitters between neurons and astrocytes [58]. The pathogenetic mechanism of hypomyelination remains unresolved but may involve primary axonal degeneration or impaired myelin synthesis by oligodendrocytes resulting from reduced neuronal production of aspartate and N-acetyl aspartate [58].

Furthermore, functional studies in mice have shown that disruption of *Slc25a12* results in delayed development, early postnatal lethality, hypomyelination, and neurofilament accumulation, indicating defects in axonal transport and progressive neurodegeneration [59–64]. These abnormalities have been attributed to impaired mitochondrial function, reduced ATP production, and altered neuronal structure [63]. Homozygous *Slc25a12*-mutant mice exhibit a wide range of neurological signs, including tremors, impaired coordination and balance, limb grasping, abnormal gait, seizures, and altered neurotransmitter levels (e.g., decreased GABA and serotonin, abnormal dopamine metabolism) [59–64]. Importantly, they also display CNS abnormalities such as abnormal brain morphology and enlarged lateral ventricles, features consistent with disrupted brain development and fluid accumulation (MGI 3603462) [64]. Given that the herein identified bovine *SLC25A12* missense variant was predicted to be deleterious by multiple in silico tools, we speculate that it may impair the function of the mitochondrial aspartate/glutamate antiporter, disrupting the malate-aspartate shuttle. This likely contributes to mitochondrial dysfunction, impaired redox balance, and reduced ATP generation, ultimately leading to the structural and neurological deficits observed in the affected calves. Taken together, these findings support causality of the identified variant for this congenital form of porencephaly and suggest that the diagnosed features may be part of a neurodevelopmental disorder associated with mitochondrial dysfunction and impaired neuronal energy homeostasis.

One remaining question is why there are such profound differences in the clinical and pathological phenotypes of AGC1 dysfunction across different species. The calves in this study exhibit established brain pathology and clinical signs from the point of birth, whereas the conditions described in humans, mice and dogs present with clinical onset in early life. Clinical signs and pathology in our case, as humans with DEE39 and experimental work in knockout mice, are focussed on the developing central nervous system, but the conditions described in dogs primarily involve an inflammatory myopathy. Evaluation of muscle samples from two affected Limousin calves showed equivocal evidence of myofiber degeneration, but these might be secondary to increased time spent in recumbency or denervation. There was no evidence of muscle inflammation, as described in Dutch Shepherds. Hypomyelination of the CNS is a reasonably consistent feature of DEE39 in children and is observed in the *aralar*-knockout mouse model, but significant hypomyelination was not observed in the affected calves. These differences in the clinical and pathological phenotypes may be explained by the differential impact of variants on AGC1 function, or by differences in the tissue and cell expression of AGC1, other solute carriers, or alternative metabolic pathways that could sustain mitochondrial respiration and offset the effects of AGC1 dysfunction. Further work is needed to clarify this, but the findings of this investigation yield interesting new insights into the impact of *SLC25A12* variants across species.

It is unclear whether the genetic disorder identified in the calves is also responsible for further reproductive problems that have been reported anecdotally in some of the study herds. One affected calf was aborted in the third trimester, and it is possible that other *SLC25A12* homozygous individuals were aborted or suffered early embryonic loss and resorption and so were not available for examination. Such a pattern, involving variable expressivity of a recessive disorder caused by variants in nuclear genes encoding mitochondrial proteins has recently been described for a frameshift insertion in *NOA1* in the Montbéliarde breed (OMIA 002874–9913) [65]. Initially detected through a genome-wide scan for cryptic recessive alleles associated with increased juvenile mortality in apparently healthy animals genotyped for genomic evaluation using homozygous haplotype enrichment/depletion analysis, this variant was later found to also cause congenital malformations and reduced fertility in at-risk matings between carrier parents or between carrier sires and daughters of carrier grandsires [40, 65].

Population screening of the *SLC25A12* variant revealed important insights into its distribution and potential origin. Analysis of a global WGS-cohort of representative of the genetic diversity within the bovine species, including data from the 1000 Bull Genomes Project and, French and Swiss resequencing projects, showed that this variant is extremely rare and breed-specific. To better understand its distribution within the Limousin breed, the variant was further examined in three European Limousin cohorts. Notably, the British Limousin cohort, opportunistically sampled from farms with reported cases, exhibited a relatively high allele frequency. In contrast, the Swiss cohort showed no presence of the variant, while the French cohort presented a very low allele frequency, with no homozygous animals identified and only 13 heterozygotes among 19,857 animals. Interestingly most of these carriers were related to the paternal granddam of the French Limousin bull “Sy”, who was born 25 years ago and is the common ancestor of the parents of the affected calves with available pedigree records.

This supports the hypothesis of a recessively inherited disorder with a local founder effect, underlining the importance of regional genetic surveillance to manage and prevent the spread of deleterious alleles within breeding programs [66, 67].

A limitation of this study is the lack of direct functional assays confirming the impact of the identified *SLC25A12* variant in the encoded protein. The liposomal transport function assays of the defective gene product, as previously described [68], and biochemical analysis of levels of lactate in serum and aspartate in brain tissue were not possible to performed. Additionally, the relatively small and non-representative cohort of British Limousin controls may limit the generalizability of these findings within the population of UK. Further screening of the *SLC25A12* variant in larger and more diverse cohorts, as well as investigation of its potential impact on embryonic lethality, is warranted in future studies.

## Conclusions

We describe the first naturally occurring *SLC25A12*-related recessively inherited form of porencephaly in a mammalian species. The clinical, pathological and genetic evidence, supported by comparative data from other mammalian species, suggests that disruption to mitochondrial aspartate/glutamate transport and energy metabolism is the underlying pathogenic mechanism. Identifying this variant expands the spectrum of *SLC25A12*-related disorders observed in different species, emphasising the gene’s consistent role in neurodevelopment and mitochondrial function. Notably, affected Limousin calves may represent a valuable spontaneous large animal model for studying *SLC25A12*-associated neurodevelopmental disorders and mitochondrial dysfunction. Furthermore, the fact that the emergence of this defect is due to a founder effect in British Limousin, highlights the need for targeted genetic surveillance to limit the spread of this deleterious allele within breeding programs. Finally, while testing for common viruses remains essential when investigating potentially teratogenic congenital malformations in aborted, stillborn, or live-born calves, the possibility of an underlying genetic cause should also be considered.

## Supplementary Information

Below is the link to the electronic supplementary material.

Additional file 1.

Additional file 2.

Additional file 3.

Additional file 4.

Additional file 5.

Additional file 6.

## Data Availability

All Swiss WGS data is available on the European Nucleotide archive under the Swiss Comparative Bovine Resequencing Project accession number PRJEB83441. The individual sample accession numbers of all cases and sire used in the current study are: case 1, ERS22569949; case 2, ERS22569948; case 3, ERS22569947; sire, ERS22569946. French WGS data are also publicly available, as previously reported previously [40–42] and is presented in Additional File 2.
